# Prevalence of potentially harmful multidrug interactions on medication lists of elderly ambulatory patients

**DOI:** 10.1186/s12877-021-02594-z

**Published:** 2021-11-19

**Authors:** Tara V. Anand, Brendan K. Wallace, Herbert S. Chase

**Affiliations:** 1 Department of Biomedical informatics, Columbia University Medical Center, 622 West 168th Street, New York, NY 10032 USA; 2 Vagelos College of Physicians and Surgeons, Columbia University, New York, NY 10032 USA

**Keywords:** Multidrug interactions, Drug-drug interactions, DDIs, Polypharmacy, Adverse drug events, ADEs

## Abstract

**Background:**

It has been hypothesized that polypharmacy may increase the frequency of multidrug interactions (MDIs) where one drug interacts with two or more other drugs, amplifying the risk of associated adverse drug events (ADEs). The main objective of this study was to determine the prevalence of MDIs in medication lists of elderly ambulatory patients and to identify the medications most commonly involved in MDIs that amplify the risk of ADEs.

**Methods:**

Medication lists stored in the electronic health record (EHR) of 6,545 outpatients ≥60 years old were extracted from the enterprise data warehouse. Network analysis identified patients with three or more interacting medications from their medication lists. Potentially harmful interactions were identified from the enterprise drug-drug interaction alerting system. MDIs were considered to amplify the risk if interactions could increase the probability of ADEs.

**Results:**

MDIs were identified in 1.3 % of the medication lists, the majority of which involved three interacting drugs (75.6 %) while the remainder involved four (15.6 %) or five or more (8.9 %) interacting drugs. The average number of medications on the lists was 3.1 ± 2.3 in patients with no drug interactions and 8.6 ± 3.4 in patients with MDIs. The prevalence of MDIs on medication lists was greater than 10 % in patients prescribed bupropion, tramadol, trazodone, cyclobenzaprine, fluoxetine, ondansetron, or quetiapine and greater than 20 % in patients prescribed amiodarone or methotrexate. All MDIs were potentially risk-amplifying due to pharmacodynamic interactions, where three or more medications were associated with the same ADE, or pharmacokinetic, where two or more drugs reduced the metabolism of a third drug. The most common drugs involved in MDIs were psychotropic, comprising 35.1 % of all drugs involved. The most common serious potential ADEs associated with the interactions were serotonin syndrome, seizures, prolonged QT interval and bleeding.

**Conclusions:**

An identifiable number of medications, the majority of which are psychotropic, may be involved in MDIs in elderly ambulatory patients which may amplify the risk of serious ADEs. To mitigate the risk, providers will need to pay special attention to the overlapping drug-drug interactions which result in MDIs.

**Supplementary Information:**

The online version contains supplementary material available at 10.1186/s12877-021-02594-z.

## Background

Elderly patients may suffer from several chronic conditions that benefit from targeted pharmacologic therapy. Consequently, there has been a steady rise in the number of medications these patients take daily, which increases the likelihood of adverse drug events (ADEs) [1, 2]. Drug-drug interactions (DDIs) are an increasingly common cause of morbidity and mortality in the elderly accounting for nearly 5 % of hospital admissions from the emergency room [3–6].

The steady increase in polypharmacy, where patients are prescribed five or more medications, also raises the risk of multidrug interactions (MDIs) in which one (or more) drugs interact with two or more other drugs, amplifying the probability of a patient experiencing an ADE [2, 7]. Amplification can result in a number of ways, including when three or more medications cause the same ADE (pharmacodynamic), when two or more drugs reduce the metabolism of a third medication (pharmacokinetic), or when one drug increases the susceptibility to ADEs associated with two other medications by altering a patient’s physiological state (conditional). While current clinical decision support in electronic health records (EHR) assign risk severity levels to DDIs, these values may underestimate the true risk for patients exposed to MDIs.

The objectives of our study were to identify the frequency of MDIs in the medication lists of ambulatory elderly patients as well as the most common drugs and ADEs associated with them.

## Methods

### Source of patient data

We identified 46,997 patients who were actively followed in the outpatient clinics of our institution from 2015 to 2019 and whose age was ≥ 60 years. The most recent medication list for each patient was extracted and used for analysis. We then excluded patients whose lists contained medications generally prescribed to hospitalized patients (enoxaparin, vancomycin, neomycin, dalteparin, lactulose or heparin), assuming that these patients had been recently hospitalized and that their medication lists had not yet been updated. Some of the medications were listed by brand names, which required conversion to generic names. Topical and ophthalmic preparations were excluded from the list of medications. Research involving human data was performed in accordance with the Declaration of Helsinki and was approved by the University’s Institutional Review Board.

All medications on the lists were associated with a date on which the medication was either prescribed or entered by providers updating the list of the patient’s medications. The dates of the medication entries on lists ranged from all medications entered on the same day to ten years or more separating the oldest and newest entries. We considered that some of the medications that had been entered years ago should have been removed and remained on the list because of inadequate medication reconciliation. To minimize the likelihood that some medications no longer belonged on the list, we chose to analyze only those medication lists in which all medications had been entered or recorded on the same day. The final number of unique patients, whose individual list was analyzed, was 6,545.

### Identification of multidrug interactions

Our institution utilizes a two drug-drug interaction decision support tool (Allscripts) that consults a list of DDIs from a Cerner Multum table modified by our institution [8]. We developed a network analysis method, which involves representing drugs and interactions graphically, to identify more complex MDIs, as two-way drug-drug interaction pairs would not identify overlapping DDIs [9]. The steps involved are described in the Supplementary Fig. 1.

### Amplification

**Fig. 1:**
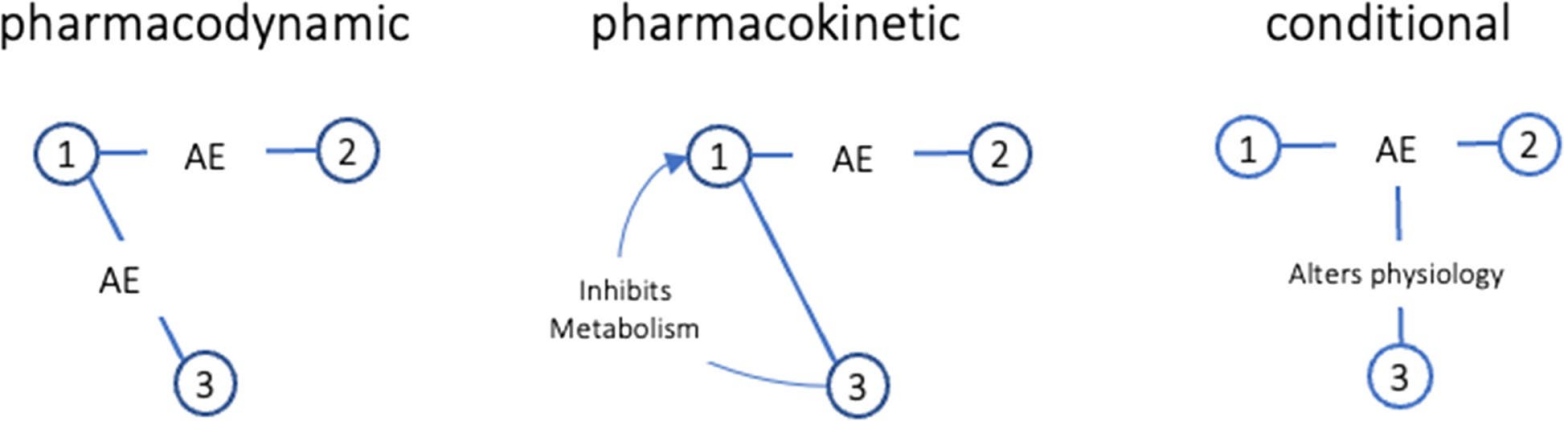
Three ways one drug interacting with two other drugs might increase the probability of an adverse event associated with a drug-drug interaction

Multidrug interactions were classified as amplifying if a third drug potentially increased the risk of an ADE associated with a two-drug interacting pair (Fig. 1). The third drug might have the same action as the other two (pharmacodynamic), inhibit the metabolism of one or both of the other two interacting drugs (pharmacokinetic) or amplify the effect of the other two interacting drugs by altering the patients’ physiology (conditional).

### List of potential associated ADEs

We reviewed each of the ADEs associated with the overlapping DDIs that comprised the MDI and compiled a list of the most serious ones based on the description in the Cerner database. Only interactions rated *contraindicated*, *generally avoid*, or *monitor closely* were included in the identification of the MDIs.

## Results

### Drug-drug interactions

The 6,545 patients whose medications lists were extracted for analysis had a mean age of 72.5 ± 8.2 and were 53 % female. A total of 20,755 medications, 533 of which were unique, were on the medication lists. Of these patients’ lists, 487 (7.4 %) contained two-drug DDIs, involving 1,042 medications, of which 146 were unique, and 85 (1.3 %) contained MDIs involving 305 medications, of which 116 were unique. Five of the 85 medication lists contained a second MDI bringing the total MDIs to 90. The average number of medications on the lists was 3.1 ±2.3 in patients with no drug interactions, 6.6 ± 2.9 in patients with two-drug DDIs, and 8.6 ±3.4 in patients with MDIs. The majority of MDIs in patients’ medications lists involved three interacting drugs (75.6 %) while the remainder were composed of four (15.6 %) or five or more (8.9 %) interacting drugs.

### Medications involved in multidrug interactions

The most common medications involved in the MDIs and their associated drug classes are listed in Table 1. Psychotropic medications were the most involved representing 35.1 % of all drugs associated with MDIs. Medications affecting the cardiovascular system and hemostasis, and opiates were the other major classes involved.

**Table 1:** Most common drugs involved in MDIs per class

Drugs in MDIs by class (% all drugs)	Class (% of all drugs)
bupropion (6.6)	Psychotropic (35.1)
trazodone (4.3)	
escitalopram (3.6)	
sertraline (3.0)	
fluoxetine (2.3)	
amiodarone (3.0)	Cardiovascular (11.1)
diltiazem (1.3)	
tramadol (4.6)	Opiate (9.8)
oxycodone (2.3)	
aspirin (3.6)	Hemostasis (8.9)
warfarin (1.6)	
clopidogrel (1.6)	
apixaban (1.3)	
methotrexate (3.0)	Immunosuppressant (8.2)
ibuprofen (1.3)	NSAIDs (4.3)
cyclobenzaprine (2.6)	Muscle relaxant (3.0)

Although the overall prevalence of MDIs was 1.3 %, the prevalence of MDIs in patients prescribed one of a small subset of drugs was considerably higher (Table 2). The highest prevalence of MDIs was on medication lists containing amiodarone (27.3 %), followed by methotrexate (23.1 %), bupropion (18.7 %), tramadol (16.3 %), trazadone (14.6 %), and cyclobenzaprine (14.3 %). The numbers of Medicare beneficiaries who filled prescriptions with the highest prevalence drugs are listed on Table 2 as well as the calculated number of patients potentially exposed to MDIs [10].

**Table 2:** The prevalence of the drugs involved in MDIs. Based on prevalence, number of Medicare beneficiaries at risk for an MDI was calculated

Medication	% Involved in MDI	CMS Part D beneficiaries	Involved in MDI
amiodarone	27.3	706,029	192,746
methotrexate	23.1	527,799	121,922
bupropion	18.7	1,714,050	320,527
tramadol	16.3	4,266,058	695,367
trazodone	14.6	2,915,625	425,681
cyclobenzaprine	14.3	1,860,548	266,058
fluoxetine	11.5	1,334,205	153,434
ondansetron	10.5	3,153,351	331,102
quetiapine	10.3	1,247,664	128,509
citalopram	9.8	1,684,688	165,099
mirtazapine	8.3	1,345,516	111,678
escitalopram	7.7	2,007,721	154,595
sertraline	7.1	2,683,062	190,497
venlafaxine	6.7	1,075,483	72,057
oxycodone	5.9	4,050,823	238,999
**TOTAL**		30,572,622	3,568,272
**TOTAL psychotropics**		16,009,014	1,722,078

### Adverse drug events associated with MDIs

**Fig. 2:**
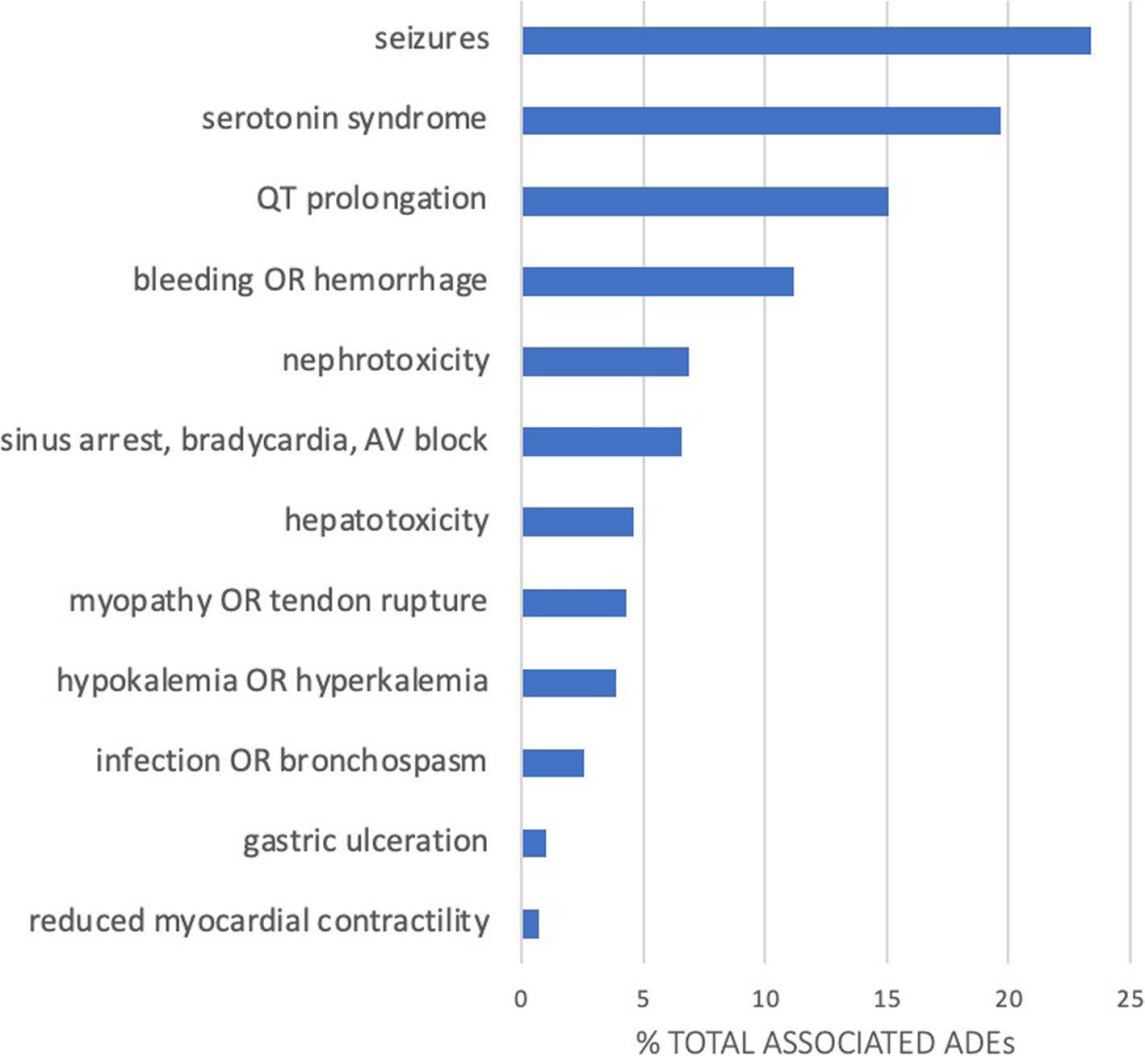
Frequency of potential adverse drug events (ADEs) associated with MDIs

The most common potential ADEs associated with the MDIs involved the central nervous system (seizures and serotonin syndrome) representing 43.1 % of the total ADEs (Fig. 2). Those ADEs associated with psychotropic medications (prolonged QT, seizures, serotonin syndrome) represented 58.2 % of all potential ADEs resulting from MDIs. Many patients with MDIs were exposed to two or more potential ADEs, the most common of which were seizures and serotonin syndrome, seizures and prolonged QT, and prolonged QT and serotonin syndrome. ADEs associated with cardiovascular system (prolonged QT, sinus arrest, AV block and bradycardia), comprised 22.5 % of the total potential ADEs. Drug interactions that predisposed patients to bleeding or hemorrhage (hemostasis) comprised 11.2 % of the ADEs.

### Amplification

Examples of how the risk of ADEs might be amplified in MDIs are reported in Supplementary Table 1. Of the 90 MDIs on the 85 medication lists, all but four were potentially amplifying resulting from one or more of the three basic mechanisms (see Fig. 1): pharmacodynamic (patients A, B, C); pharmacokinetic (patient D); and conditional (patient E). All three mechanisms were involved in patient F.

### Severity of the interactions

 The MULTUM categorization of severity of the individual overlapping drug-drug pairs that comprised the MDIs varied from *contraindicated* (2.1 %), *generally avoid* (27.4 %) or *monitor closely* (70.5 %). The severity of each of the interacting drug-drug pairs that comprise the MDI are listed in Supplementary Table 1. Inasmuch as MDIs are composed of two or more overlapping drug-drug interactions, each with a specific severity rating, the rating of an MDI is a composite of the individual severities which ranged from [*contraindicated – contraindicated*] interactions (patient A ) to [*monitor closely – monitor closely – monitor closely – generally avoid*] (patient F).

## Discussion

### Multidrug interactions

Adverse drug reactions have been estimated by the FDA to be the fourth leading cause of death in the US resulting in costs of over $500 billion [11, 12]. Drug-drug interactions increase the likelihood of ADEs either because both drugs have the same target or effect, or one drug inhibits the metabolism of the other. It follows that the probability of patients experiencing ADEs would be further amplified if three or more drugs interacted with each other. We found that 1.3 % of the 6,545 medication lists of a cohort of elderly ambulatory patients contained MDIs. The prevalence of MDIs in medication lists was considerably higher in those that included one of a subset of medications; MDIs were identified in more than 20 % of the medication lists containing amiodarone or methotrexate and more than 10 % of lists containing bupropion, tramadol, trazodone, cyclobenzaprine, fluoxetine, ondansetron, or quetiapine (Table 2).

### Common medications and amplification of ADEs

 Medications for psychiatric conditions were the medications most frequently involved in MDIs, accounting for 35.1 % of all medications involved, a finding consistent with several prior studies of two-drug interacting pairs [13–20]. Given the frequency of psychotropic medications in MDIs, the most common potential serious ADEs were those associated with psychotropic medications, namely prolonged QT, seizures, and serotonin syndrome [21–23].

Nearly all MDIs could amplify the risk of patients experiencing associated ADEs. There are two predominant mechanisms of amplification. First, there are pharmacodynamic additive effects of medications such as prolonging the QT interval, lowering the seizure threshold, or causing the serotonin syndrome [23–27]. Second, there were pharmacokinetic additive effects in which multiple interactions could result in steep increases in the concentration of participating medications resulting from CYP inhibition. Sutherland et al. found that 4 % of elderly patients in the cohort they studied were taking multiple inhibitors of the same CYP enzyme [4]. Psychotropic medications, which were the most common class of medications represented in MDIs from our study, operate by both mechanisms to amplify the risk of serotonin syndrome, seizures, and torsades de pointes [28, 29]. Patient F (Supplementary Table 1) is likely to have significantly elevated levels of the psychotropic mirtazapine resulting from the inhibition of its metabolism by both bupropion and fluoxetine and the inhibition of fluoxetine’s metabolism by bupropion [22, 28, 30–32]. Age-related changes in pharmacokinetics due to diminished hepatic and renal function in the elderly make pharmacokinetic interactions especially likely [33].

### Relationship between actual and potential ADEs resulting from MDIs

 Prior studies of DDIs found that between 6 % and 20 % of patients with DDIs identified in their records experienced an associated ADE [17, 20, 34–36]. While there are as yet no studies to ascertain what proportion of patients exposed to MDIs experience an ADE, it is likely that the number will exceed that observed in two-drug interactions given the amplifying effect of additional interacting drugs. For example, patients taking warfarin and aspirin are more likely to have a hemorrhagic episode if they are also taking amiodarone. Polypharmacy has been associated with increased bleeding risk in patients on warfarin, even after adjusting for confounding factors [37]. We observed that the average number of medications on the lists of patients with MDIs was considerably higher (8.6 ±3.4) than those without (3.1 ± 3.4).

Prospective studies of ambulatory patients who experience ADEs due to multidrug interactions will be challenging because ADEs experienced outside of the hospital may be unaccounted for if the patient is admitted to another healthcare facility or suffers a lethal reaction. We note that an increased rate of sudden death associated with psychotropic medications has been observed [38–45]. The FDA adverse event reporting system (FAERS) has recently been mined to identify multidrug interactions and the resulting ADEs actually experienced by patients [46]. While this approach cannot determine accurately the prevalence of ADEs associated with MDIs, it can establish that MDIs are associated with documented serious ADEs. In the absence of prospective studies, an examination of medication lists provides an opportunity to estimate the potential for amplification of the risk of ADEs.

### Risk of ADEs in Medicare beneficiaries

 In 2019, approximately 30 million CMS Part D Medicare beneficiaries were prescribed one of the top 15 medications involved in the MDIs we identified. Based on the prevalence we observed for MDIs in patients prescribed these drugs, approximately 3.5 million patients could have been exposed to amplifying MDIs (Table 2) [10]. Psychotropic medications were prescribed to nearly 16 million CMS Part D Medicare beneficiaries of whom 1.6 million were potentially exposed to amplifying MDIs. The use of amiodarone is particularly worrisome because, in addition to its effects on the lung, liver, and thyroid, it inhibits several key CYP enzymes (1A2, 2C9, 2D6, 3A4) and prolongs the QT interval [47, 48]. The number of CMS Part D Medicare beneficiaries prescribed amiodarone in 2019 was 706,029 of which 192,746 patients were potentially exposed to serious MDIs. Assuming that, on average, 10 % of patients with drug interactions experience the associated ADEs, approximately 20,000 patients taking amiodarone could have experienced a serious ADE [17, 20, 34–36].

### Alerting providers to potential MDIs

Most EHRs alert providers of potential drug-drug interactions by posting a warning of the potential ADE associated with the pair and the severity of the interaction. A patient prescribed warfarin, amiodarone and aspirin would trigger EHR warnings of two potential interactions, [amiodarone-warfarin] and [aspirin-warfarin], on separate lines along with the severity of each of the interactions. The amplifying effect of amiodarone on the warfarin-aspirin interaction would not be factored into the severity because there is no reported interaction between aspirin and amiodarone. Considering the amplifying effect of amiodarone on the warfarin-aspirin interaction, the combined severity of the drug interactions should probably be categorized as *contraindicated*. The Multum warnings of *monitor closely* for the [amiodarone-warfarin] pair and *generally avoid* for the [aspirin-warfarin] pair would underestimate the potential risk for hemorrhage (Patient D, Supplementary Table 1).

An EHR warning system that recognizes MDIs could be incorporated into the drug-drug interaction decision support but would likely result in alert fatigue that often results in providers overriding the alerts. Providers’ ignoring the alerts has been shown to increase the number of ADEs, at great cost to patients and the healthcare system [49, 50]. One solution would be to post the warning only when high-risk drugs such as psychotropics or amiodarone are involved. Until acceptable interventions are implemented, providers will need to look for multiple overlapping drug-drug interactions which share a common drug (and thus constitute an MDI).

### Strategies to reduce risk

 Once potential MDIs are recognized, providers could substitute one of the interacting drugs with a more benign, non-interacting one [51]. Amiodarone could be replaced with propafenone which would eliminate the amplified risk of hemorrhage resulting from the warfarin-aspirin interaction. The FDA and the American Geriatrics Society 2019 updated the AGS Beers Criteria® for Potentially Inappropriate Medication Use in Older Adults recommend that amiodarone should not be a first-line choice [52, 53]. Ondansetron, which prolongs the QT, could be avoided in patients already on medications that prolong the QT if prescribed anti-emetics that are not associated with changes in the QT interval [54]. Tramadol, which acts as both an opiate and inhibitor of neurotransmitter uptake, exposes patients to multiple serious ADEs. Non-opiate analgesics could be substituted for tramadol and, if there was no indication for the psychotropic effect, no additional medication would be necessary. Substitution of methotrexate, an immunosuppressant prescribed for patients with various autoimmune diseases, would be difficult to replace given its unique role in treating patients with autoimmune diseases. Other strategies would have to be employed such as meticulous attention to those medications which interact with methotrexate to increase the risk of renal or hepatic injury.

Deprescribing rather than substitution may be a more effective strategy, especially for patients on psychotropics [55]. It is not uncommon for patients to be prescribed three or more psychotropic medications, likely reflecting the increasing prevalence of psychotropic polypharmacy [56]. Given the amplifying effect of psychotropic drugs, it is essential to assess the necessity and suitability of their use in elderly patients [57–61].Trazadone, one of the most prescribed psychotropic medications participating in multidrug interactions (Table 2) and associated with serotonin syndrome and seizures, is one of the least efficacious medications in treating depression and is associated with a higher suicide rate than other psychotropic medications [62–66]. Despite the warnings, nearly 3 million Medicare beneficiaries received prescriptions for trazodone in 2019 of which over 400,000 would be expected to be exposed to an MDI including that drug (Table 2). Cyclobenzaprine, despite the characterization by the AGS Beers Criteria® for Potentially Inappropriate Medication Use in Older Adults as “questionable,” is contraindicated in patients already taking drugs that prolong the QT, cause serotonin syndrome, or cause seizures [53]. Nevertheless, 1.8 million Medicare beneficiaries were prescribed cyclobenzaprine in 2017, potentially exposing recipients to increased risk of life-threatening ADEs. Although deprescribing these medications might be the best strategy, to do so would require primary care physicians to coordinate care with the specialists who originally prescribed the other medications which is often a significant challenge [67].

### Strengths

 In 2015 Roughead warned that polypharmacy would inevitably result in multidrug interactions which would lead to an increased risk of ADEs [7]. Since that call to arms, however, there have been no studies prior to ours that identify MDIs. This may because doing so requires a more complex computational approach. To identify two-drug DDIs, drug pairs on the patients’ medication lists can be directly compared and matched to known two-drug interactions found in an enterprise EHR such as Cerner’s. Identification of MDIs, however, requires either network analysis or complex structured queries of the institutional database (Supplemental Fig. 1). Both approaches, however, are well known to clinical informaticians and could easily be applied in clinical studies. Perhaps our results will inspire other to identify and characterize MDIs in their institutions’ patient populations to expand the growing list of MDIs.

### Limitations

 There are several limitations to this study. First, we did not take into consideration the doses of the medications that were involved in the MDIs. It is possible that patients were prescribed lower doses of a particular medication than recommended because a second or third drug could influence its metabolism, thus mitigating any potentially amplifying interaction. Second, we cannot say if the medications on the list were intended for daily use or only intermittent use, as needed. Aspirin, which appeared on many lists of patients with MDIs, could have been intended to be taken daily for cardioprotective effects or only as necessary for intermittent pain. Daily aspirin would pose greater risk of an ADE, such as gastric hemorrhage, than if taken only intermittently. Third, identification of three and four MDIs is based on the drug-drug interaction tables at our institution. The identification and rating of various interactions might differ across institutions given that there is significant variability in the drug-drug interaction databases [68–73].

## Conclusions

Our results demonstrate that patient medication lists contain combinations of drugs that can participate in multidrug interactions and increase the risk of associated ADEs. The discovery of previously unrecognized drug interactions through data mining will likely increase the identification of MDIs [74–76]. Until the EHR-based drug-drug interaction warning tools recognize MDIs, providers will have to be on the lookout for overlapping interacting drug-drug pairs which constitute MDIs on the patients’ medication lists. In either case, substitution of another medication or deprescribing may be warranted to lower the risk of ADEs.

## Supplementary Information

**Additional file 1: Supplementary Figure 1.** Schematic of method used to identify potential multidrug interactions (MDIs) and associated adverse drug events (ADEs).

**Additional file 2: Supplementary Table 1.** Examples of amplifying multidrug interactions and the mechanisms involved and severity of the interactions.

## Data Availability

Due to HIPPA restrictions, supporting data cannot be made openly available. For further information about the data and conditions for access please contact Herbert S. Chase directly via email: hc15@cumc.columbia.edu.

## Abbreviations

MDIs: multiple drug interactions
DDIs: drug-drug interactions
ADE(s): adverse drug event (s)
EHR: electronic health record
CYP: cytochrome P450 enzyme
