# Supplementary figures and images for: Prevalence of potentially harmful multidrug interactions on medication lists of elderly ambulatory patients
Source: BMC Geriatr. 2021 Nov 19;21:648. doi: 10.1186/s12877-021-02594-z (PMC8603594; doi:10.1186/s12877-021-02594-z)

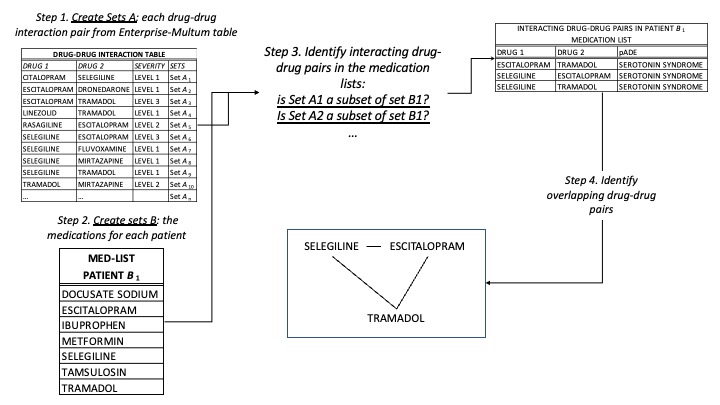

Supplement: Supplementary file 1 — Additional file 1: Supplementary Figure 1. Schematic of method used to identify potential multidrug interactions (MDIs) and associated adverse drug events (ADEs). [file 12877_2021_2594_MOESM1_ESM.jpg]
