# Supplementary material for: Prevalence of potentially harmful multidrug interactions on medication lists of elderly ambulatory patients
Source: BMC Geriatr. 2021 Nov 19;21:648. doi: 10.1186/s12877-021-02594-z (PMC8603594; doi:10.1186/s12877-021-02594-z)
Supplement: Supplementary file 2 — Additional file 2: Supplementary Table 1. Examples of amplifying multidrug interactions and the mechanisms involved and severity of the interactions. [file 12877_2021_2594_MOESM2_ESM.docx]

Supplementary Table 1

**Supplementary Table 1** Examples of amplifying multidrug interactions and the mechanisms involved and severity of the interactions.

| Patient | Medication list drugs | drug-drug pairs | ADE | SEV | Comments |
| --- | --- | --- | --- | --- | --- |
| A | aspirin ketorolac meloxicam | aspirin-ketorolac | HEM, RF, ULC | 1 | PD: pairs associated with same AE |
|  |  | ketorolac-meloxicam |  | 1 |  |
| B | tramadol  duloxetine trazodone | tramadol-duloxetine, | SZ, SS | 2 | PD: pairs associated with two different AE |
|  |  | duloxetine-trazodone | SS | 3 |  |
|  |  | tramadol-trazodone | SZ, SS | 2 |  |
| C | tramadol  amitriptyline  donepezil amantadine | tramadol-amitriptyline | SZ | 3 | PD: one drug (tramadol) involved in four interactions associated with same AE |
|  |  | tramadol-donepezil |  | 3 |  |
|  |  | tramadol-amantadine |  | 3 |  |
|  |  | tramadol-hydrocodone |  | 2 |  |
| D | amiodarone warfarin  aspirin | amiodarone-warfarin | HEM | 3 | PK: Amiodarone inhibits warfarin metabolism; warfarin and aspirin are associated with hemorrhage |
|  |  | warfarin-aspirin |  | 2 |  |
| E | torsemide amiodarone sertraline | torsemide-amiodarone | pQT | 2 | COND: Torsemide reduces K+ increasing risk of pQT |
|  |  | amiodarone-sertraline |  | 2 |  |
| F | fluoxetine mirtazapine  bupropion | fluoxetine-mirtazapine | SS | 3 | PK: bupropion inhibits mirtazapine and fluoxetine metabolism.  PD: fluoxetine and mirtazapine associated same AE.  COND: fluoxetine and mirtazapine lower seizure threshold |
|  |  | mirtazapine-bupropion | SZ | 3 |  |
|  |  | bupropion-fluoxetine |  | 3 |  |
|  |  | amiodarone-sertraline |  | 2 |  |

Legend: Mechanisms: pharmacodynamic (PD); Pharmacokinetic (PK); conditional (COND). Associated adverse events: serotonin syndrome (SS); prolonged QT interval (pQT); seizures (SZ), hemorrhage (HEM), renal failure (RF), ulceration (ULC). Severity (SEV): 1 = contraindicated; 2 = generally avoid; 3 = monitor closely.
